# Infra-acetabular screw exited between ischial tuberosity and ischial spine is more suitable for Asian population: a 3D morphometric study

**DOI:** 10.1186/s12891-020-03802-4

**Published:** 2020-11-28

**Authors:** Fei Liu, Xiaoreng Feng, Yang Xiao, Jie Xiang, Keyu Chen, Yihang Deng, Jiaxin Lv, Bin Chen

**Affiliations:** 1 Division of Orthopaedics and Traumatology, Department of Orthopaedics, Nanfang Hospital, Southern Medical University, No. 1838 North Guangzhou Avenue, 510515 Guangzhou, China; 2 Department of Orthopaedics and Traumatology, Yangjiang people’s Hospital, Yangjiang, 529535 China

**Keywords:** Infra-acetabular screw, Bone corridor, Asian population, 3D-reconstruction

## Abstract

**Background:**

Recently, the infra-acetabular screw has been proposed for use in treatment of acetabular fractures as a part of a periacetabular fixation frame. Biomechanical studies have shown that an additional infra-acetabular screw placement can enhance the fixation strength of acetabular fracture internal fixation. Currently, the reported exit point of the infra-acetabular screw has been located at the ischial tuberosity (Screw I). However, our significant experience in placement of the infra-acetabular screw has suggested that when the exit point is located between the ischial tuberosity and the ischial spine (Screw II), the placement of a 3.5 mm infra-acetabular screw may be easier for some patients. We conducted this study in order to determine the anatomical differences between the two different IACs.

**Methods:**

The raw datasets were reconstructed into 3D models using the software MIMICS. Then, the models, in the STL format model, were imported into the software Geomagic Studio to delete the inner triangular patches. Additionally, the STL format image processed by Geomagic Studio was imported again into MIMICS. Finally, we used an axial perspective based on 3D models in order to study the anatomical parameters of the two infra-acetabular screw corridors with different exit points. Hence, we placed the largest diameter virtual screw in the two different screw corridors. The data obtained from this study presents the maximum diameter, length, direction, and distances between the entry point and center of IPE.

**Results:**

In 65.31% males and 40.54% females, we found a screw I corridor with a diameter of at least 5 mm, while a screw II corridor was present in 77.55% in males and 62.16% in females. Compared to screw I, the length of screw II is reduced, the angle with the coronal plane is significantly reduced, and the angle with the transverse plane is significantly increased.

**Conclusions:**

For East Asians, changing the exit point of the infra-acetabular screw can increase the scope of infra-acetabular screw use, especially for females.

## Background

The acetabulum has a complex anatomical structure and is surrounded by some important blood vessels, nerves, and organs. The acetabular fracture surgery is the most complex and challenging surgery in the field of traumatic orthopedics [1]. Hence, patients with acetabular fractures may face many post-surgical complications, including traumatic osteoarthritis, deep vein thrombosis, and heterotopic ossification [1, 2]. Conducting a good fracture reduction and strong internal fixation can allow postoperative patients to be able to perform functional rehabilitation exercises earlier, thereby reducing the occurrence of related complications and achieving the best clinical effect [1].

In 2010, the infra-acetabular screw was reported to be able to help close the incomplete “frame” system around the acetabulum, including both acetabular columns, supra-acetabular screws, and the true pelvic rim plate [3]. Meanwhile, researchers indicated the range of application of infra-acetabular screw, including acetabular fractures, that require ilioinguinal approach treatment. The column separation caused by the fracture line passing through the acetabular quadrilateral, as well as obturator foramen, are indications [3]. Specifically, the indication includes anterior column fracture, double-column fracture, T-type fracture, and anterior and posterior hemitransverse fracture. A biomechanical study showed that the locking plate internal fixation system cannot significantly reduce the displacement of high anterior column fractures after fixation. However, no matter what kind of internal fixation was used, adding an infra-acetabular screw can approximately double the strength of the internal fixation [4]. The same fracture pattern was used for another biomechanical study, in which three groups of screws of different materials (titanium, stainless steel, degradable materials) were compared to the general plate. The results indicated that the screw fixation could be applied to non-comminuted acetabular anterior column fractures. The screw fixation had a fixation strength that was equivalent to that of the general plate fixation, which is a promising alternative to steel plate fixation. Moreover, the use of an infra-acetabular screw can significantly increase the strength of fracture fixation, regardless of the implant type [5]. Clinical application has demonstrated that the infra-acetabular screw can achieve good results, including good reduction, fewer complications, and lower risk of postoperative displacement [6, 7].

Despite the fact that the infra-acetabular screw has several advantages, its disadvantages include that it is difficult to place the screw as the screw corridor is relatively narrow and it is an unconventional screw corridor. Some researchers believe that some patients are not suitable for infra-acetabular screws [8]. Therefore, in order to provide references for successful placement of the infra-acetabular screws, many scholars have studied the diameter, length, entry point, spatial position and spatial shape of infra-acetabular corridor (IAC). An anatomical study of 523 pelvises using computer-aided technology showed that 93% of specimens had an IAC with a diameter of lower than 5 mm [8]. A morphological research based on CT scanning demonstrated that the IAC always presents a biconical shape and the narrowest part is located in the fovea of the acetabulum [2]. In addition, development the dysplasia hip does not adversely affect infra-acetabular screw placement. The perfect insertion point of the infra-acetabular screw is highly consistent, and the distance between the entry point and the caudal and medial sides of the ilio-pubic / ilio-pectineal eminence (IPE) center is 10.2 mm and 10.4 mm respectively, which provides a reference for the rapid insertion of infra-acetabular screw through the intrapelvic approach for treatment of related acetabular fractures [9]. The results of these researchers have given surgeons a comprehensive understanding of the anatomical characteristics of the infra-acetabular screw, which can greatly help the rapid and accurate placement of screws during an operation. However, the specimens used by these researchers are from Europe and North America, which means the research results are not applicable to East Asians due to the differences in bone structure between the two races.

In this study, the exit points of the infra-acetabular screw have been located in the ischial tuberosity [2, 6, 8–10]. For Chinese patients that have acetabular fractures, we often cannot successfully insert an infra-acetabular screw. Therefore, we used computer-aided technology to study the structure around the acetabulum and found that when the exit point is located between the ischial tuberosity and the ischial spine, there is also an IAC. This discovery may provide an additional option for IAC. However, at present, there are limited reports on the anatomical characteristics of this new IAC. Therefore, we conducted a purely anatomical study to determine the anatomical differences between the two different IACs. We hypothesized that when the screw exit point is between the ischial spine and ischial tuberosity, the IAC has a larger diameter, which helps increase the range of infra-acetabular screws that can be utilized in East Asian populations represented by Chinese.

## Methods

### Data collection

All of the procedures were conducted in accordance with the declaration of Helsinki and relevant policies in China. The study protocol was granted approval by the ethics review board at Nanfang Hospital, Southern Medical University. We obtained verbal consent from all subjects prior to collecting data for this. Overall, 86 subjects that were admitted to our institution without pelvic and acetabular injury or lesions were recruited in this study (Table 1). All subjects underwent a 16-line pelvic helical computed tomography scan (GE, US) with 1.0-mm slices at 0.1-s intervals for imaging of the acetabulum. Pelvic CT scans without any evidence of pelvic tumor or deformity were included. The raw data obtained were stored in Dicom format.

**Table 1:** Characteristics of pelvis specimens

Variable	Pelves	Females	Males	Significance
Number in overall data set	86	37	49	
Mean age [95% CI] (years)	49.90[3.42]	47.08[5.17]	52.02[4.63]	NS
Range of age (years)	19–86	20–73	19–86	
Mean body height [95% CI] (cm)	163.15[1.48]	158.24[1.61]	166.85[1.68]	*P* < 0.001
Range of body height (cm)	146–178	146–173	150–178	
Mean body weight [95% CI] (kg)	58.15[2.18]	53.01[2.63]	62.03[2.89]	*P* < 0.001
Range of body weight (kg)	39–82.5	39–72	41–82.5	

*CI* Confidence interval, *NS* Not significant

### Model reconstruction

The raw data sets were reconstructed into 3D models using the software MIMICS 17.0 (Materialise, Leuven, Belgium). The left hemi-pelvis was exported in STL format and imported into the image-processing software Geomagic Studio 2013 (Geomagic, US). Next, the inner triangular patches, which represented contents of the marrow cavity, were deleted in order to make the marrow cavity hollow in 3D models. After processing in Geomagic Studio 2013, the images were exported in STL format and imported again into MIMICS where all the simulations and measurements were carried out.

### Largest screw path analysis

In recent years, application of computer-aided technology has allowed the rapid development of digital orthopedics. A bio-morphological study of screw corridors around the acetabulum can be performed easily and quickly using computer-aided techniques [11–13]. Our team has proposed using an “axial perspective” to study the largest anterior and posterior column screw corridors of the acetabulum on the axial view [12, 14]. Therefore, in this study, an “axial perspective” was used to study the anatomical parameters of the two different IACs.

**Fig. 1:**
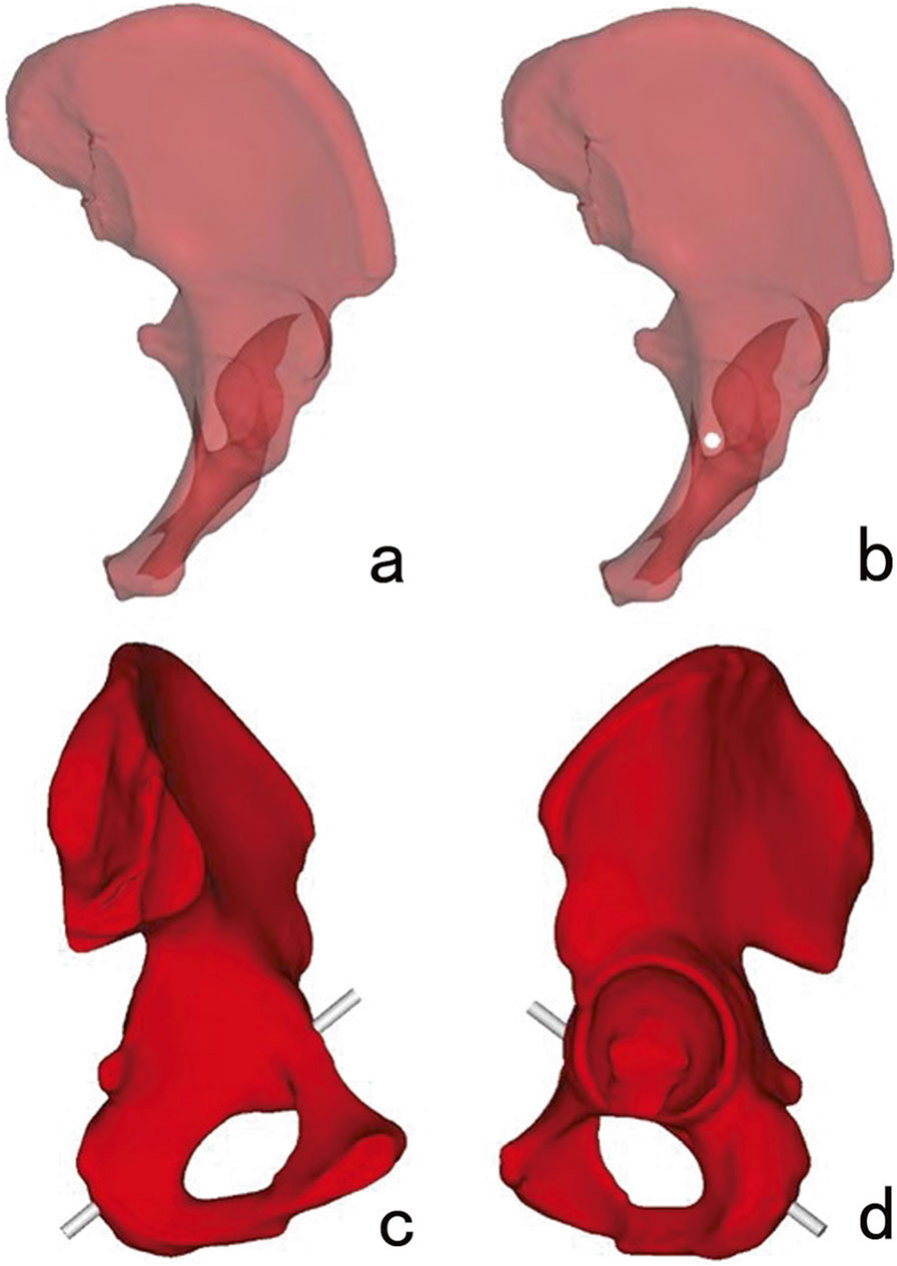
Placement of Screw I. **a** The 3D model was adjusted to identify the largest ellipse-like translucent area. **b** A virtual screw was placed in the translucent area. **c**, **d** The position of Screw I was validated in the opaque 3D model

**Fig. 2:**
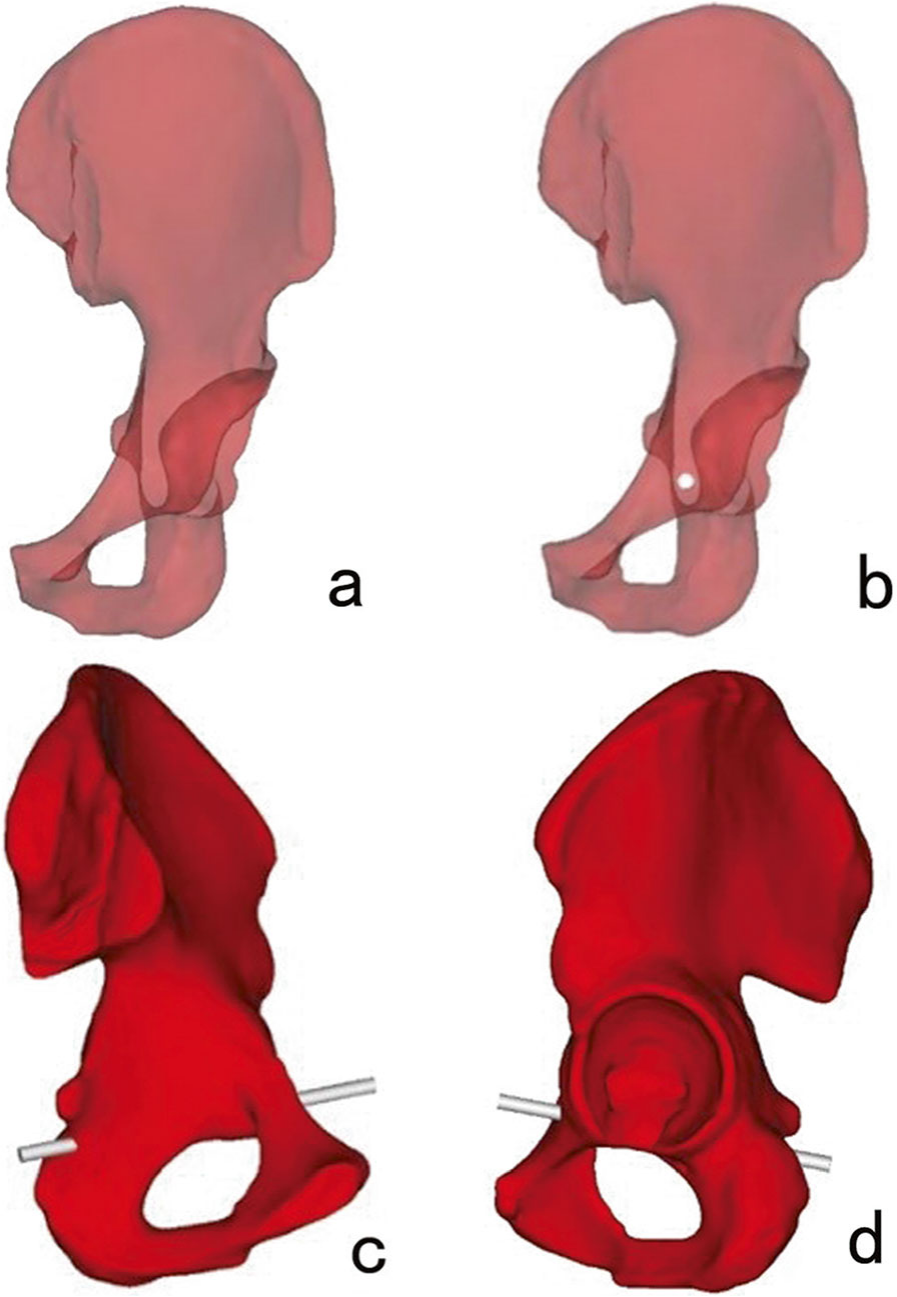
Placement of Screw II. **a** The 3D model was adjusted to identify the largest ellipse-like translucent area. **b** A virtual screw was located in the translucent area. **c**, **d** The position of Screw II was validated in the opaque 3D model

In order to distinguish the screw path in 3D models from the same perspective, we downgraded the transparency of 3D models and turned 3D models at the axial perspective of the infra-acetabular corridor, which is a view that is perpendicular to the cross-section of the two infra-acetabular corridor axis. As a result, a translucent area with a darker outline of each infra-acetabular corridor was clearly seen. The translucent area represented the marrow cavity of the infra-acetabular corridor, the outline of which showed the cortical bone (Figs. 1 and 2).

First, we adjusted positions of the 3D model in the axial perspective in order to find the most likely largest translucent area by visual observation. Then, a virtual computer-aided design (CAD) Screw I (2 mm in diameter) was placed perpendicular on the screen at the center of the translucent area. We adjusted the CAD Screw I diameter progressively in order to accurately determine the maximum implant diameter that the transparent area could accommodate. The maximum implant diameter was defined as the largest diameter of the screw that did not penetrate the outline of the translucent area (Figs. 1 and 2). After these steps were repeated across the three most likely largest transparent areas by three observers, we obtained three accurate maximum implant diameters. A comparison was conducted in order to finalize the accurate maximum implant diameter of the screw that the specific 3D model could accommodate. The maximum screw diameters, the caudal and medial distances of the entry point to the center of IPE, and the directions of the screw to the transverse, coronal and sagittal planes were measured, respectively. The same procedure was applied to screw II.

### Statistical analysis

All experimental data (continuous variables) were presented as mean and standard deviation (SD) or median and range. An independent sample t-test was utilized to compare data between males and females. Paired sample t-test was used to compare the data between Screw I and Screw II. Linear regression was used to analyze the linear relationships between the two different infra-acetabular parameters and age, body height, and body weight. Statistical significance was set at *p* < 0.05. The SPSS statistical software package for Windows (version 19.0) was used for all statistical analyses.

## Results

Among 65.31% (32/49) males and 40.54% (15/37) females, we found a Screw I corridor with a diameter of at least 5 mm. The mean maximum diameter and length of Screw I were 5.30 ± 1.25 mm (2.62–7.58 mm) and 94.57 ± 6.07 mm (78.99–105.91 mm), respectively. The caudal and medial distances of the entry point to the center of IPE of Screw I were 7.49 ± 4.68 mm (− 6.72–21.43 mm) and 12.32 ± 3.12 mm (5.66–23.17 mm), respectively. The angles of Screw I to the transverse, coronal and sagittal planes were 36.48 ± 8.18° (15.95–54.38°), 52.77 ± 8.14° (35.50–72.52°), − 0.49 ± 6.43° (− 18.04–11.20°), respectively. All differences, with the exception of the coronal angle of the Screw I between males and females, were statistically significant (Table 2).

**Table 2:** Differences between males and females

Group	Diameter (mm)		Length(mm)		Anterior(mm)		Medial(mm)		Sagittal (°)		Coronal(°)		Transverse(°)	
	SI	SII	SI	SII	SI	SII	SI	SII	SI	SII	SI	SII	SI	SII
Males	5.63 ± 1.23	6.02 ± 1.36	98.14 ± 4.24	81.05 ± 4.55	6.27 ± 4.11	20.55 ± 4.98	11.20 ± 2.70	11.26 ± 2.60	4.16 ± 2.79	3.86 ± 2.17	51.28 ± 6.61	76.04 ± 6.71	38.30 ± 6.48	12.78 ± 7.11
Females	4.85 ± 1.14	5.23 ± 1.32	89.84 ± 4.74	78.99 ± 4.11	9.12 ± 4.94	19.81 ± 5.05	13.79 ± 3.06	13.94 ± 3.26	−6.65 ± 4.32	−7.88 ± 5.05	54.74 ± 9.55	74.10 ± 7.76	34.08 ± 9.56	12.58 ± 8.01
t	3.039	2.702	8.551	2.176	2.920	0.679	4.154	4.235	13.290	13.260	1.888	1.242	2.313	0.122
p	0.003	0.008	0.000	0.032	0.004	0.499	0.000	0.000	0.000	0.000	0.064	0.218	0.024	0.903

Among 77.55% (38/49) males and 62.16% (23/37) females, we found a screw II corridor with a diameter of at least 5 mm. The mean maximum diameter and length of Screw II were 5.68 ± 1.39 mm (2.56–8.26 mm) and 80.16 ± 4.46 mm (71.17–89.78 mm), respectively. The caudal and medial distance of the entry point to the center of IPE of Screw II was 20.24 ± 5.00 mm (9.67–32.35 mm) and 12.41 ± 3.18 mm (6.09–21.01 mm), respectively. The angles of Screw II to the transverse, coronal and sagittal planes were 12.69 ± 7.46°(0.03–34.19°), 75.20 ± 7.20°(51.33–88.98°), − 1.19 ± 6.91°(− 17.26–8.86°), respectively. The differences in diameter, length, medial distance and angles to the sagittal planes of Screw II between males and females were statistically significant (Table 2), but differences of caudal distance, angles to the transverse and coronal planes of the Screw II between males and females were not statistically significant (Table 2).

In both males and females, differences in diameter, length, anterior distance, angles to transverse and coronal planes between Screw I and Screw II were statistically significant (Table 2). However, the differences of medial distance and angles to the sagittal plane between Screw I and Screw II were not statistically significant (Table 3). Additionally, body height and body weight were strongly correlated with the length of the two different infra-acetabular corridors in males (*r* > 0.3; *p* < 0.05) (Tables 4 and 5).

**Table 3:** Differences between Screws I and Screw II

Group	Diameter (mm)		Length (mm)		Anterior (mm)		Medial (mm)		Sagittal(°)		Coronal(°)		Transverse(°)	
	M	F	M	F	M	F	M	F	M	F	M	F	M	F
SI	5.63 ± 1.23	4.85 ± 1.14	98.14 ± 4.24	89.84 ± 4.74	6.27 ± 4.11	9.12 ± 4.94	11.20 ± 2.70	13.79 ± 3.06	4.16 ± 2.79	−6.65 ± 4.32	51.28 ± 6.61	54.74 ± 9.55	38.30 ± 6.48	34.08 ± 9.56
SII	6.02 ± 1.36	5.23 ± 1.32	81.05 ± 4.55	78.99 ± 4.11	20.55 ± 4.98	19.81 ± 5.05	11.26 ± 2.60	13.94 ± 3.26	3.86 ± 2.17	−7.88 ± 5.05	76.04 ± 6.71	74.10 ± 7.76	12.78 ± 7.11	12.58 ± 8.01
t	5.128	4.316	35.447	18.808	21.312	15.860	0.211	0.304	0.776	1.968	23.597	23.353	23.831	20.819
p	0.000	0.000	0.000	0.000	0.000	0.000	0.834	0.763	0.442	0.057	0.000	0.000	0.000	0.000

*SI* Screw I, *SII* Screw II

**Table 4:** Correlation analysis of anthropometric and parameters of Screw I

Parameter	Age		Body height		Body weight	
	*r* value	*p* value	*r* value	*p* value	*r* value	*p* value
Females						
Diameter	0.190	0.259	0.124	0.464	0.018	0.914
Length	0.311	0.061	0.240	0.152	0.275	0.099
Anterior	0.085	0.616	0.024	0.889	0.155	0.361
Medial	0.078	0.645	0.051	0.766	0.061	0.719
Sagittal	0.296	0.076	0.014	0.936	0.001	0.994
Coronal	0.088	0.605	0.110	0.517	0.126	0.457
Transverse	0.053	0.754	0.100	0.558	0.112	0.510
Males						
Diameter	0.034	0.819	0.115	0.431	0.089	0.542
Length	0.104	0.477	**0.647**	**5.15E-7**	**0.610**	**3.23E-6**
Anterior	0.272	0.058	0.217	0.135	0.137	0.346
Medial	0.066	0.650	0.170	0.244	0.085	0.561
Sagittal	0.233	0.108	0.112	0.444	0.058	0.694
Coronal	0.130	0.372	0.226	0.119	0.078	0.596
Transverse	0.121	0.406	0.227	0.116	0.077	0.599

**Table 5:** Correlation analysis of anthropometric and parameters of Screw II

Parameter	Age		Body height		Body weight	
	*r* value	*p* value	*r* value	*p* value	*r* value	*p* value
Females						
Diameter	0.218	0.194	0.067	0.692	0.007	0.965
Length	0.044	0.796	0.269	0.107	0.326	0.049
Anterior	0.066	0.696	0.079	0.644	0.155	0.360
Medial	0.076	0.654	0.251	0.133	0.041	0.809
Sagittal	0.383	0.019	0.180	0.286	0.030	0.860
Coronal	0.048	0.779	0.100	0.558	0.053	0.755
Transverse	0.101	0.551	0.135	0.425	0.024	0.888
Males						
Diameter	0.027	0.856	0.042	0.776	0.019	0.897
Length	0.274	0.057	**0.507**	**2.01E-4**	**0.467**	**0.001**
Anterior	0.124	0.395	0.073	0.620	0.009	0.949
Medial	0.193	0.185	0.028	0.848	0.044	0.766
Sagittal	0.004	0.977	0.078	0.594	0.014	0.924
Coronal	0.127	0.386	0.042	0.772	0.127	0.383
Transverse	0.111	0.448	0.038	0.795	0.144	0.322

## Discussion

The value of an infra-acetabular screw is that it is an important part of the periacetabular fixation “frame”. Biomechanical studies demonstrated that an additional placement of this screw significantly increases the strength of a standard plate fixation for treatment of anterior column fractures [4, 5]. A biomorphometric CT-based analysis was performed to study the sex-specific differences of the infra-acetabular corridor [8]. In this study, the exit point of an infra-acetabular screw was located at ischial tuberosity, where the average diameter and length of the infra-acetabular corridor were 7.4 mm (2.8–12.9 mm) and 103 mm (81–122 mm) respectively. Meanwhile, the authors found that 90% females and 94% of males have an infra-acetabular corridor that is larger than 5 mm in diameter. The 5 mm diameter was chosen to be a threshold in infra-acetabular corridor anatomical studies as a corridor with a diameter of at least 5 mm was defined as a threshold for placement of a 3.5 mm cortical screw in clinical practice [8]. However, in our study, when the exit point of the infra-acetabular screw was at IPE, the average diameter and length of the infra-acetabular screw were 5.30 mm (2.62–7.58 mm) and 94.57 mm (78.99–105.91 mm), respectively. Furthermore, only 40.54% of females and 65.31% of males had an infra-acetabular corridor with a diameter of at least 5 mm. The differences between these two studies may be caused by racial differences.

At present, in studies of the infra-acetabular screw, the exit point of the screw was located in the ischial tuberosity. However, our study identified that when the exit point of the screw was located between ischial tuberosity and the ischial spine, there is also an IAC. According to our results, a screw II corridor with a diameter of at least 5 mm was found in 77.55% of males and 62.16% of females, which was increased by 12.24 and 21.62% respectively compared to the Screw I corridor. That demonstrated that the Screw II corridor could increase the proportion of IAC exceeding 5 mm in Eastern population, which enables the placement of a 3.5 mm screw in some patients with a small screw I corridor, particularly in female patients. Most of the Screw II corridor diameters were increased compared to Screw I, with an average increase of 7.20%. Meanwhile, all of the Screw II corridor lengths were shortened, with an average reduction of 15.24%. Length shortening can greatly reduce the difficulty of inserting a long screw into a narrow corridor. In males, the two different infra-acetabular corridors were parallel to the sagittal plane, while angles to the sagittal plane of the two corridors of females were larger than that of males, and the directions were from the medial to the lateral side. Compared to Screw I, the entry point of Screw II is farther away caudally from the center of IPE.

Our results demonstrate that due to a narrower corridor of the infra-acetabular screw in Asian populations, some patients are not suitable for use of infra-acetabular screws. Therefore, we recommend that each patient that intends to insert an infra-acetabular screw should have a CT scan and use a digital orthopedic software to perform a preoperative virtual placement of an infra-acetabular screw prior to surgery. In this way, a personalized surgical plan can be developed for patients to improve the safety of the operation. Furthermore, intraoperative navigation and 3D printing technology have been utilized to help place periacetabular screws [10, 15–17], which can be used to help successfully place an infra-acetabular screw.

This was a purely anatomical study that utilized computer-aided technology. Therefore, there were certain unavoidable limitations. CT resolution, the adopted smoothing criteria, selection of seed threshold and sensitivity can all cause the 3D model to not conform to the real specimen. Furthermore, observer errors can cause observation and measurement errors. The sample size may not be enough to represent the entire population. The absence of a correlation between the values found and additional morphometric pelvic parameters can cause preoperative planning of the two different infra-acetabular screws to rely more on CT scans. More importantly, this study lacks clinical support. In the future, clinical research needs to be conducted in order to account for this limitation.

## Conclusion

Our study provided surgeons with the anatomical parameters of two different IACs in an Eastern population. Compared to previous research, we found that the proportion of Screw I with a diameter of at least 5 mm was greatly reduced, while Screw II could reduce this gap. This may provide an alternative acetabular infra-acetabular screw placement corridor for surgeons.

## Data Availability

All data used in this article were collected from raw CT data of 86 subjects. The dataset in this study is available from the corresponding author upon request.

## Abbreviations

IAC: Infra-acetabular screw corridor
CT: Computed tomography
3D: Three-dimensional reconstruction
IPE: Ilio-pubic / ilio-pectineal eminence
Mimics: Materialise’s Interactive Medical Image Control System
CAD: Computer-aided design
SPSS: Statistical Product and Service Solutions
